# Janus Kinase Inhibitor Baricitinib Modulates Human Innate and Adaptive Immune System

**DOI:** 10.3389/fimmu.2018.01510

**Published:** 2018-06-28

**Authors:** Satoshi Kubo, Shingo Nakayamada, Kei Sakata, Yukihiro Kitanaga, Xiaoxue Ma, Seunghyun Lee, Akina Ishii, Kaoru Yamagata, Kazuhisa Nakano, Yoshiya Tanaka

**Affiliations:** ^1^The First Department of Internal Medicine, University of Occupational and Environmental Health, Kitakyushu, Japan; ^2^Mitsubishi Tanabe Pharma, Yokohama, Japan; ^3^Astellas Pharma Inc., Tokyo, Japan; ^4^The Department of Pediatrics, The First Hospital of China Medical University, Shenyang, China

**Keywords:** baricitinib, tofacitinib, Janus kinase, signal transducer and activator of transcription, human immunology

## Abstract

The purpose of this study was to elucidate the mechanism of action of baricitinib on Janus kinase (JAK)/signal transducer and activator of transcription (STAT) signaling, which involves in human innate and adaptive immune system. The effects of baricitinib were evaluated using human monocyte-derived dendritic cells (MoDCs), plasmacytoid dendritic cells (pDCs), B cells, and T cells. Baricitinib concentration-dependently suppressed the expression of CD80/CD86 on MoDCs and the production of type-I interferon (IFN) by pDCs. Baricitinib also suppressed the differentiation of human B cells into plasmablasts by B cell receptor and type-I IFN stimuli and inhibited the production of interleukin (IL)-6 from B cells. Human CD4^+^ T cells proliferated after T cell receptor stimulation with anti-CD3 and anti-CD28 antibody; however, such proliferation was suppressed by baricitinib in a concentration-dependent manner. In addition, baricitinib inhibited Th1 differentiation after IL-12 stimulation and Th17 differentiation by TGF-β1, IL-6, IL-1β, and IL-23 stimulation. Tofacitinib showed similar effects in these experiments. In naive CD4^+^ T cells, IFN-α and IFN-γ induced phosphorylation of STAT1, which was inhibited by baricitinib and tofacitinib. Furthermore, IL-6-induced phosphorylation of STAT1 and STAT3 was also inhibited by JAK inhibitors. In conclusion, the results indicated that baricitinib suppresses the differentiation of plasmablasts, Th1 and Th17 cells, as well as innate immunity, such as the T cell stimulatory capacity of dendritic cells. Thus, JAK inhibitors can be potentially clinically effective not only in rheumatoid arthritis but other immune-related diseases.

## Introduction

The therapeutic strategies for rheumatoid arthritis (RA) are described in detail in the treat-to-target RA recommendations (1, 2). Inhibitors that target Janus kinase (JAK), a tyrosine kinase, were introduced as targeting synthetic disease-modifying antirheumatic drugs (tsDMARDs) in the 2016 updated recommendation (3). Since tsDMARDs are low-molecular weight compounds (molecular weight less than 1,000 kDa), they can be administered *via* the oral route, whereas biological DMARDs (bDMARDs) require intravenous or subcutaneous injection. Moreover, because they are easily delivered into the cells, they can directly inhibit the target intracellular signaling molecules.

Janus kinases, which constitutively bind to cytokine receptors, play an important role in the cytokine signaling pathways. While JAKs are comprised of JAK1, JAK2, JAK3, and tyrosine kinase-2 (Tyk2), more than 40 types of cytokines transmit signals through JAKs (4). Tofacitinib, which selectively targets JAK1 and JAK3 (5, 6) and has subsequently been found to inhibit JAK2 (7), is reported to be highly effective in the treatment of RA (8–12). This finding accelerated the development of other JAK inhibitors. After several clinical studies, baricitinib, a highly selective inhibitor of JAK1 and JAK2 (13), has been approved recently for the treatment of RA (14–17) in Europe, Japan, and other countries. Although this drug is available orally due to its small molecular weight, it has comparable efficacy to the bDMARDs (17).

One of the major goals of research in the field of human immunology is to develop highly specific molecular targeting drugs that can inhibit specific molecules in human immune cells. Since it has been so far relatively difficult to produce complete functional loss of a single molecule in human cells, unlike in mice, it is difficult to investigate how a particular molecule affects the human immune networks. Thus, the advent of highly specific molecular target drugs will facilitate the elucidation of the significance of JAKs in human immunology, especially because this subject cannot be studied in mice. In fact, differences between mice and humans have been highlighted in several studies on autoimmune diseases, and the results of many aspects of studies conducted in mice cannot be extrapolated to humans, especially the more complex human immune network. For example, in the treatment of systemic lupus erythematosus, resistance to conventional immunosuppressants develops due to the immunological heterogeneity in this disease (18, 19). Thus, realizing the pathological complexities of human autoimmune diseases, we need to expand our understanding of the complex human immune networks, including various types of immune cells and cytokines.

The availability of various selective JAK inhibitors allows analysis of the roles of JAKs in human immune responses. We reported previously that through its selective inhibition of JAK1 and JAK3, tofacitinib inhibits lymphocyte proliferation and production of cytokines (20), and that it affects the maturation of human monocyte-derived dendritic cells (MoDCs) and their capacity to stimulate T cells (21). Based on these results, it appears that JAKs have great significance in the immune networks of both innate and adaptive immunity. This study was designed to determine the effects of a highly selective JAK1 and JAK2 inhibitor, baricitinib, on human immunocompetent cells, to establish the significance of JAKs and the potential for baricitinib in the therapeutic armamentarium against immune-mediated diseases.

## Materials and Methods

### JAK Inhibitors

Baricitinib was kindly provided by Eli Lilly (Indianapolis, IN, USA). Tofacitinib was kindly provided by Pfizer (New York, NY, USA). Anti-interleukin (IL)-6 receptor α antibody, tocilizumab, was purchased from Chugai Pharmaceutical Co. (Tokyo, Japan).

### Flow Cytometric Analysis

Flow cytometric analysis was conducted as described previously (21). Briefly, the cells were incubated in blocking buffer and then suspended in FACS solution with fluorochrome-conjugated monoclonal antibodies. The cells were analyzed with a FACSVerse (Becton-Dickinson, San Jose, CA, USA) and analyzed with Flow Jo software (Tree Star, Ashland, OR, USA). Isotype-matched mouse IgG controls (BD Biosciences, Franklin Lakes, NJ, USA) were used to evaluate the background. Cell viability was evaluated by Annexin V and Propidium Iodide (BioLegend, San Diego, CA, USA).

### Generation of MoDCs and Cell Cultures

Monocyte-derived dendritic cells were generated as described in detail previously (21). Briefly, peripheral blood mononuclear cells (PBMCs) were isolated from peripheral blood samples using lymphocyte separation medium (ICN/Cappel Pharmaceuticals, Aurora, OH, USA). Monocytes were obtained from PBMCs by positive magnetic selection using anti-CD14 microbeads (Miltenyi Biotec, Bergisch Gladbach, Germany) and were cultured in the presence of IL-4 and granulocyte macrophage colony-stimulating factor for 6 days. The immature MoDCs were pretreated with JAK inhibitors for 6 h and stimulated for 48 h with lipopolysaccharide (LPS) (100 ng/ml; Sigma-Aldrich, St. Louis, MO, USA) to mature.

### Evaluation of Plasmacytoid Dendritic Cells (pDCs)

**Figure 1:**
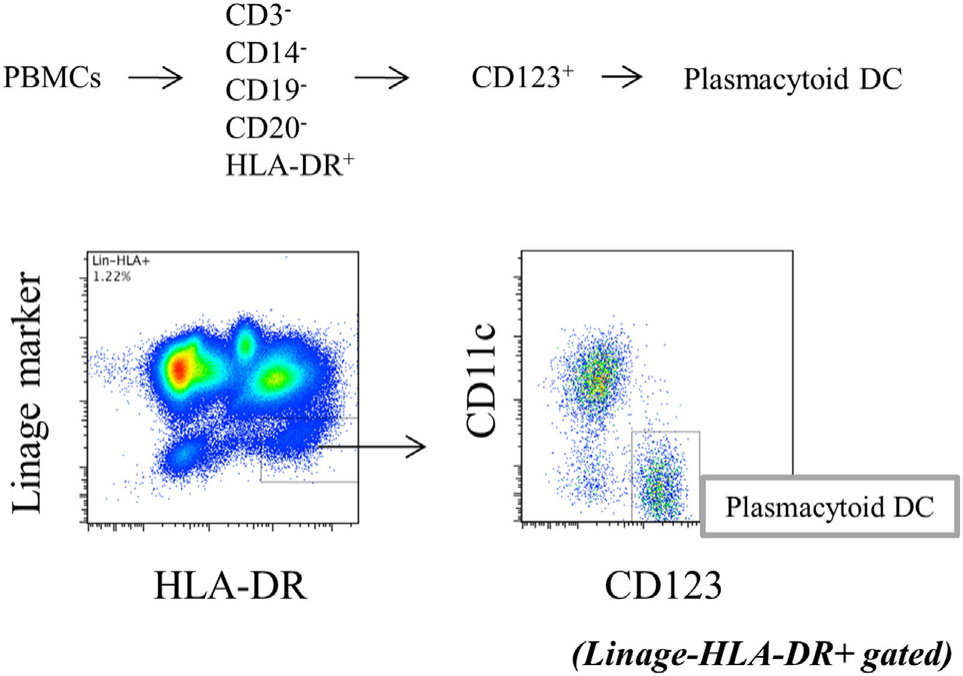
Gating strategy of plasmacytoid dendritic cells (Lin^−^HLA^−^DR^+^CD11c^−^CD123^+^).

Peripheral blood mononuclear cells (1 × 10^6^/well) in 48-well plates or pDCs (2.5 × 10^4^/well) in 96-well flat-bottom plates were stimulated with toll-like receptor (TLR) 9 agonist, CpG2216 (2 µmol/l, all from InvivoGen, San Diego, CA, USA) for 5 h, with the addition of brefeldin A (2.5 μg/mL: Sigma-Aldrich) during the final 3 h of stimulation to block cytokine secretion. Cells were stained with FITC-conjugated Lineage cocktail 1 (which includes anti-CD3: clone SK7, anti-CD14: clone MFP9, anti-CD16: clone 3G8, anti-CD19: clone SJ25C1, anti-CD20: clone L27 and anti-CD56: clone NCAM16.2), V500-conjugated anti-HLA-DR (clone G46-6), PE-Cy7-conjugated anti-CD11c (clone B-ly6), and 135 PerCP-Cy5.5-conjugated anti-CD123 (clone 7G3). After fixation and permeabilization with fixation/permeabilization buffer (eBioscience, Santa Clara, CA, USA), the cells were stained with PE-conjugated anti-interferon (IFN)-α2b (clone 7N4-1) for TLR9-mediated IFN-α production by pDCs. After intracellular staining, Lin^−^HLA-DR^+^ CD11c^−^CD123^+^ cells were gated as pDCs (Figure 1), and cytokine positivity in pDCs was used as an indicator of cytokine production by pDCs. To evaluate the cytokine production, pDCs were purified from PBMC. Primary human pDCs were purified using Diamond Plasmacytoid Dendritic Cell Isolation Kit II (Miltenyi Biotec), and purity was always >90%. IFN-α concentration in culture supernatants was measured by BD™ Cytometric Bead Array (BD Biosciences). All antibodies were purchased from BD Biosciences.

### Isolation, Culture, and Stimulation of B Cells

Human B cells were purified from whole blood by Dynabeads™ CD19 Pan B and DETACHaBEAD™ CD19 Kit (Thermo Fisher Scientific, Waltham, MA, USA). The purified B cells were resuspended (1 × 10^6^ cells/ml) in RPMI 1640 medium containing 10% FCS at 37°C and cultured for 5 days under goat anti-human IgM Ab F(ab′)2 fragments (1 µg/ml; Jackson ImmunoResearch, West Grove, PA, USA) and IFN-α (1,000 U/mL) stimulation. Differentiation of CD3^−^CD19^+^CD20^−^CD27^hi^CD38^hi^ plasmablasts was assessed by flow cytometry. IgG Ab titer and IL-6 concentration in culture supernatants were measured by Human IgG ELISA Quantitation Set (Bethyl laboratories, Montgomery, TX, USA) and BD™ Cytometric Bead Array (BD Biosciences), respectively.

### T Cell Culture

Human naive CD4^+^ T cells were purified by Naive CD4^+^ T Cell Isolation Kit II (Miltenyi Biotec) according to the protocol recommended by the manufacturer. The obtained cells were cultured in flat-bottomed 96-well plates (2 × 10^5^ cells/well) coated with anti-CD3 antibody (2 µg/ml; R&D systems, Minneapolis, MN, USA) and supplemented with soluble anti-CD28 antibody (0.5 µg/ml; R&D systems) and cytokines [IL-12 (10 ng/ml), TGF-β (1 ng/ml), IL-6 (10 ng/ml), IL-1β (5 ng/ml), and IL-23 (10 ng/ml)]. T cell proliferation was assessed by [^3^H] thymidine incorporation in the last 16 h. For Th1 differentiation, T cells were cultured with IL-12. Likewise, T cells were cultured with TGF-β, IL-6, IL-1β, IL-23, and anti-IFN-γ antibody (5 µg/ml; eBioscience) for Th17 differentiation. For intracytoplasmic staining, the cells were stimulated for 2 h with phorbol 12-myristate 13-acetate (50 ng/ml), ionomycin (1 µg/ml), and brefeldin A for 3 h, and then stained with APC-anti-IFN-γ antibody (BD Biosciences) or APC-anti-IL-17 antibody (eBioscience). The analyses were performed on living cells, which is indicated by the negative expression of Fixable Viability Dye (eBioscience). For analysis of phosflow, cells were hatched for 10 min with Phosflow Fix Buffer I and treated for 30 min at 4°C with Perm Buffer III, then stained with Alexa Fluor 647 anti-p signal transducer and activator of transcription (STAT)1 antibody (BD Biosciences), PE anti-pSTAT3 antibody (BD Biosciences), PE anti-pSTAT4 antibody (BD Biosciences), and Alexa Fluor 647 anti-pSTAT6 antibody (BD Biosciences).

### Statistical Analysis

Data of at least three independent experiments (different donors were used for the cells employed) were analyzed for the differences. The differences were examined using the Mann–Whitney test. A *p* value of <0.05 denoted the presence of a significant difference.

### Ethics Approval for the Study

This study was reviewed and approved by the ethics committee of medical research, University of Occupational and Environmental Health, Japan. PBMC were obtained from health volunteers. A signed informed consent was obtained from all subjects in accordance with the Declaration of Helsinki and its subsequent modifications.

## Results

### Baricitinib Suppresses CD80/CD86 Expression on LPS-Stimulated Human MoDCs

**Figure 2:**
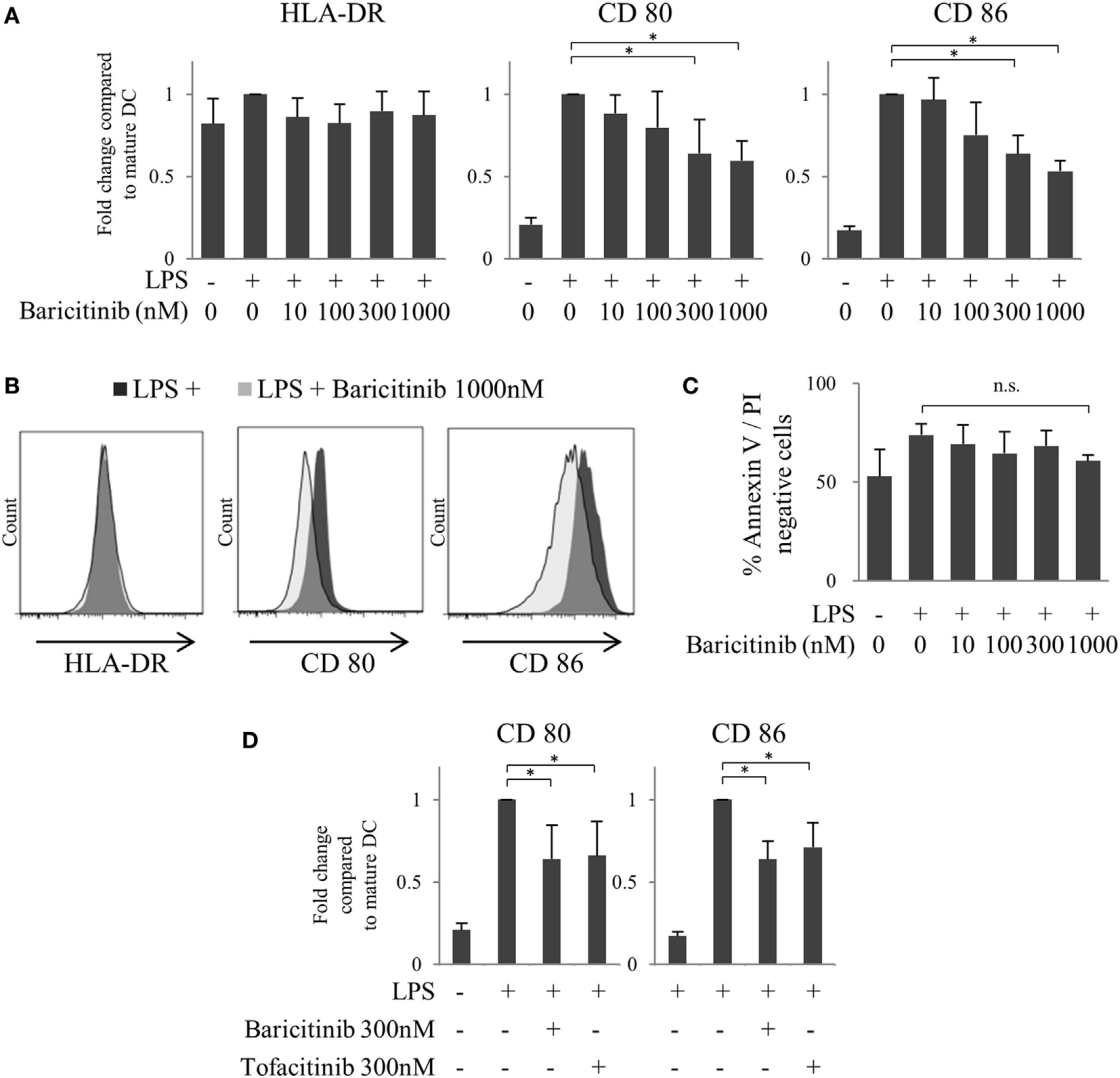
Baricitinib suppresses CD80/CD86 expression on dendritic cells (DCs). Immature monocyte-derived dendritic cells were cultured with or without baricitinib and tofacitinib during lipopolysaccharide stimulation for 48 h. The DC phenotype was evaluated using flow cytometry. **(A)** Expression of HLA-DR, CD80, and CD86. **(B)** Representative histogram data of HLA-DR, CD80, and CD86 expression. **(C)** Rate of viable cells (annexin V_neg_/propidium iodide_neg_). **(D)** Expression of CD80 and CD86 in the presence of baricitinib and tofacitinib. Data are mean ± SD of three different donors per group. *p < 0.05 and **p < 0.01 (by Student’s *t*-test).

First, we investigated the effects of JAK inhibitors on the expression of costimulators of human MoDCs. CD80/86 were induced 48 h after LPS stimulation. However, baricitinib downregulated CD80/CD86 expression, but not that of HLA-DR, in MoDCs in a concentration-dependent manner (Figures 2A,B). Although the production of TNFα and IL-6 was induced by stimulation of MoDCs with LPS for 48 h that was suppressed by baricitinib in a high concentration condition (data not shown). To examine the cytotoxicity of baricitinib on dendritic cells (DCs), the cells were stained with annexin V and propidium iodide. Baricitinib did not induce apoptosis, even at concentrations as high as 1,000 nM for 48 h (Figure 2C). These results were predicted since we reported previously that tofacitinib (a JAK1 and JAK3 inhibitor) suppressed costimulatory molecules *via* signal loop inhibition of type-I IFN and interferon regulatory factor (IRF)-7 (21). The specificity of the finding was reported previously [21]; CD80/CD86 expression was not downregulated by JAK2 inhibitors. Thus, the above results suggest that suppression of CD80/CD86 expression was dependent on inhibition of JAK1. Indeed, inhibition of CD80/CD86 expression by tofacitinib was comparable to that induced by baricitinib (Figure 2D).

### Baricitinib Inhibits Type-I IFN Production by CpG-Stimulated Plasmacytoid DCs

**Figure 3:**
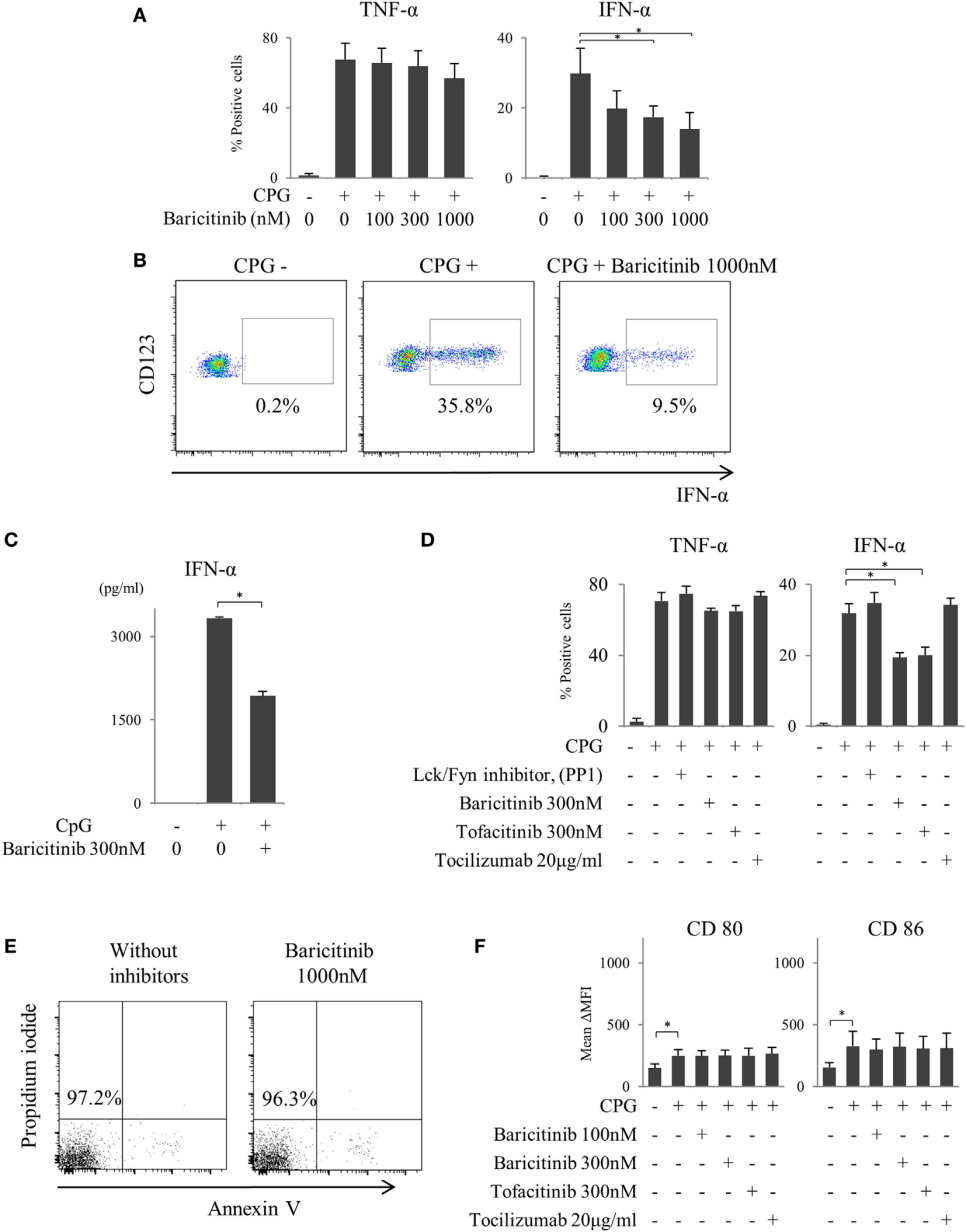
Baricitinib suppresses type-I interferon (IFN) production from plasmacytoid dendritic cells (pDCs). Peripheral blood mononuclear cells were stimulated with toll-like receptor (TLR) 9 agonist with or without baricitinib and other inhibitors. TLR9-mediated cytokine production by pDCs (Lin^−^HLA-DR^+^ CD11c^−^CD123^+^ cells) was measured by intracellular staining or ELISA. **(A)** Percentage of TNF-α- and IFN-α-producing pDCs. **(B)** Representative flow cytometry plots showing IFN-α production by pDCs. **(C)** IFN-α concentration in the supernatants. **(D)** Percentage of TNF-α- and IFN-α-producing pDCs in the presence of baricitinib and other inhibitors. **(E)** Representative histogram data of annexin V/propidium iodide staining from three independent experiments. **(F)** Expression of CD80 and CD86 on pDCs. Data are mean ± SD of three different donors per group. *p < 0.05 (by Student’s *t*-test).

We reported previously that the suppression of costimulatory molecules in MoDCs was caused by inhibition of type-I IFN signal and its autocrine loop (21). In this regard, pDCs are considered as the main source of type-I IFN *in vivo*. Next, we investigated the effect of baricitinib on pDCs. pDCs stimulated for 5 h with TLR9 (CpG) produced both TNF-α and IFN-α. Baricitinib reduced the proportion of these IFN-α producing pDCs in a concentration-dependent manner (Figures 3A,B). On the other hand, TNF-α production was not affected by baricitinib (Figure 3A). Moreover, baricitinib (Figure 3C) and tofacitinib (Figure 3D) suppressed IFN-α concentration in the supernatant of this experiment. However, tocilizumab, which inhibits IL-6 signal (22), and another tyrosine kinase inhibitor (Lck/Fyn inhibitor) did not have these effects (Figure 3D). When the isolated pDCs were stained with annexin V and propidium iodide, baricitinib did not induce apoptosis during the experiments (Figure 3E; data are representative of three independent experiments). Meanwhile, we have examined the expression of CD80/CD86 on pDCs after TLR9 stimulation. Although the role of CD80/CD86 on human pDCs is controversial, CD80/CD86 was numerically upregulated after CpG stimulation. However, the differences in activity were not obvious, and there were no inhibitory effects of JAK inhibitor and tocilizumab (Figure 3F).

### Baricitinib Inhibits Differentiation of B Cells to Plasmablasts

**Figure 4:**
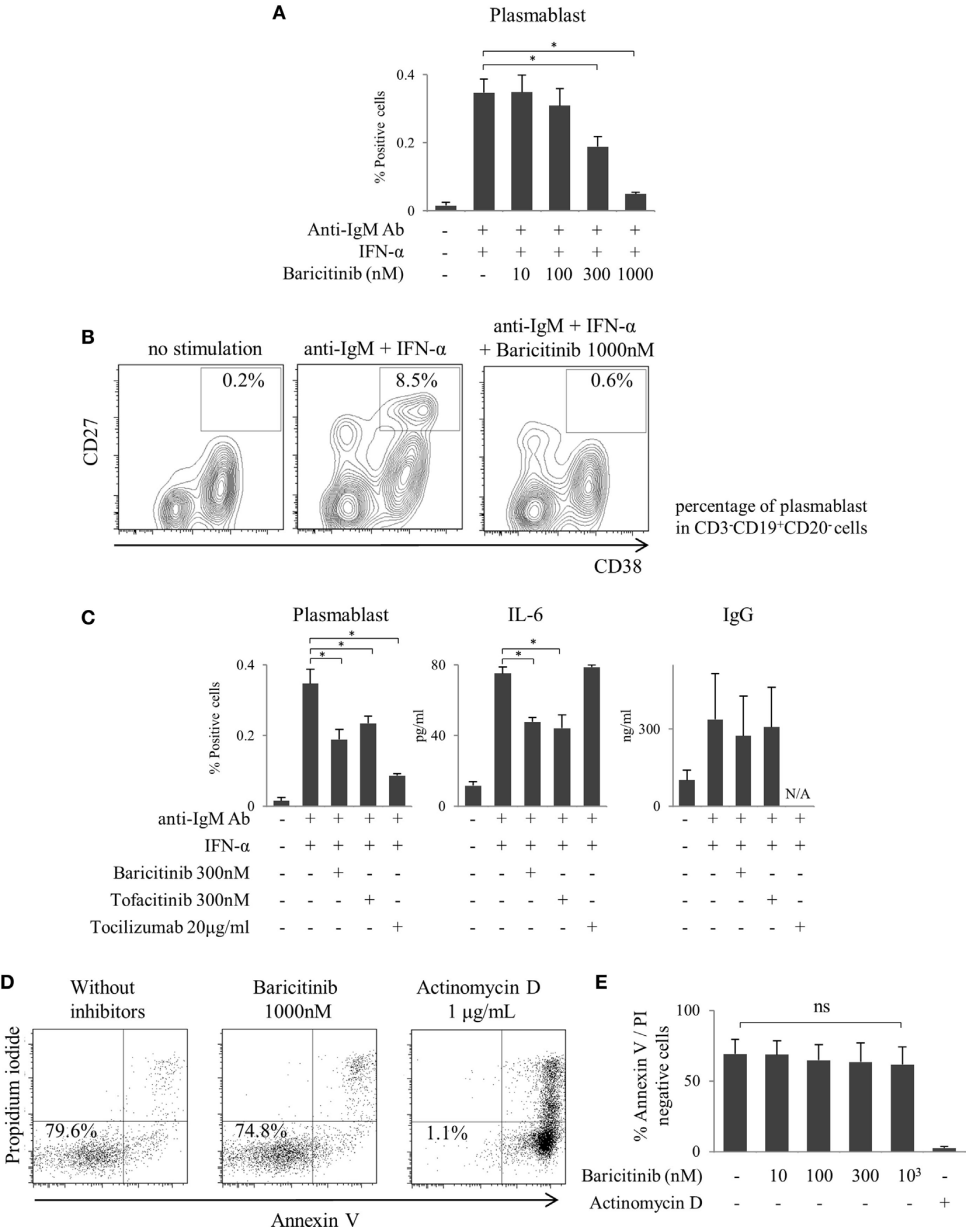
Baricitinib inhibits B cell differentiation. Human B cells were purified and cultured for 5 days in the presence of anti-human IgM Ab F(ab′)2 fragments and under interferon (IFN)-α stimulation. Plasmablast (CD3^−^CD19^+^CD20^−^CD27^hi^CD38^hi^) differentiation was assessed by flow cytometry, and IgG Ab titers and interleukin (IL)-6 concentrations in culture supernatants were measured by ELISA Cytometric Bead Array. **(A)** Percentage of plasmablasts. **(B)** Representative flow cytometry plots showing plasmablasts. **(C)** Percentage of plasmablast, IL-6, and IgG concentrations in the presence of baricitinib and other inhibitors. **(D)** Representative histogram data of annexin V/propidium iodide staining. **(E)** Rate of viable cells (annexin V_neg_/propidium iodide_neg_). Data are mean ± SD of three different donors per group. *p < 0.05 (by Mann–Whitney *U* test).

As described above, inhibition of type-I IFN signaling is one of the most important mechanisms of action of JAK inhibitors on the human immune response. In the next step, we explored the role of JAKs in human B cells differentiation. B cells were differentiated into plasmablasts by B cell receptor (BCR) and IFN-α stimulation (Figure 4A). Baricitinib inhibited this differentiation in a concentration-dependent manner (Figures 4A,B). In addition, baricitinib inhibited IL-6 production by B cells (Figure 4C). To examine the viable B cells, the cells were stained with annexin V and propidium iodide in the presence of baricitinib or actinomycin D (as a positive control). Over 95% of B cells died in the presence of actinomycin D, while baricitinib did not induce apoptosis of B cells (Figures 4D,E). Tofacitinib also suppressed BCR- and IFN-α-induced plasmablast differentiation and IL-6 production. However, neither baricitinib nor tofacitinib altered IgG production by B cells. On the other hand, inhibition of IL-6 signaling, which is important for B cell differentiation, resulted in a potent suppression of plasmablast differentiation (Figure 4C).

### Baricitinib Inhibits Differentiation of Naïve CD4^+^ T Cells Into Th1 and Th17 Cells

**Figure 5:**
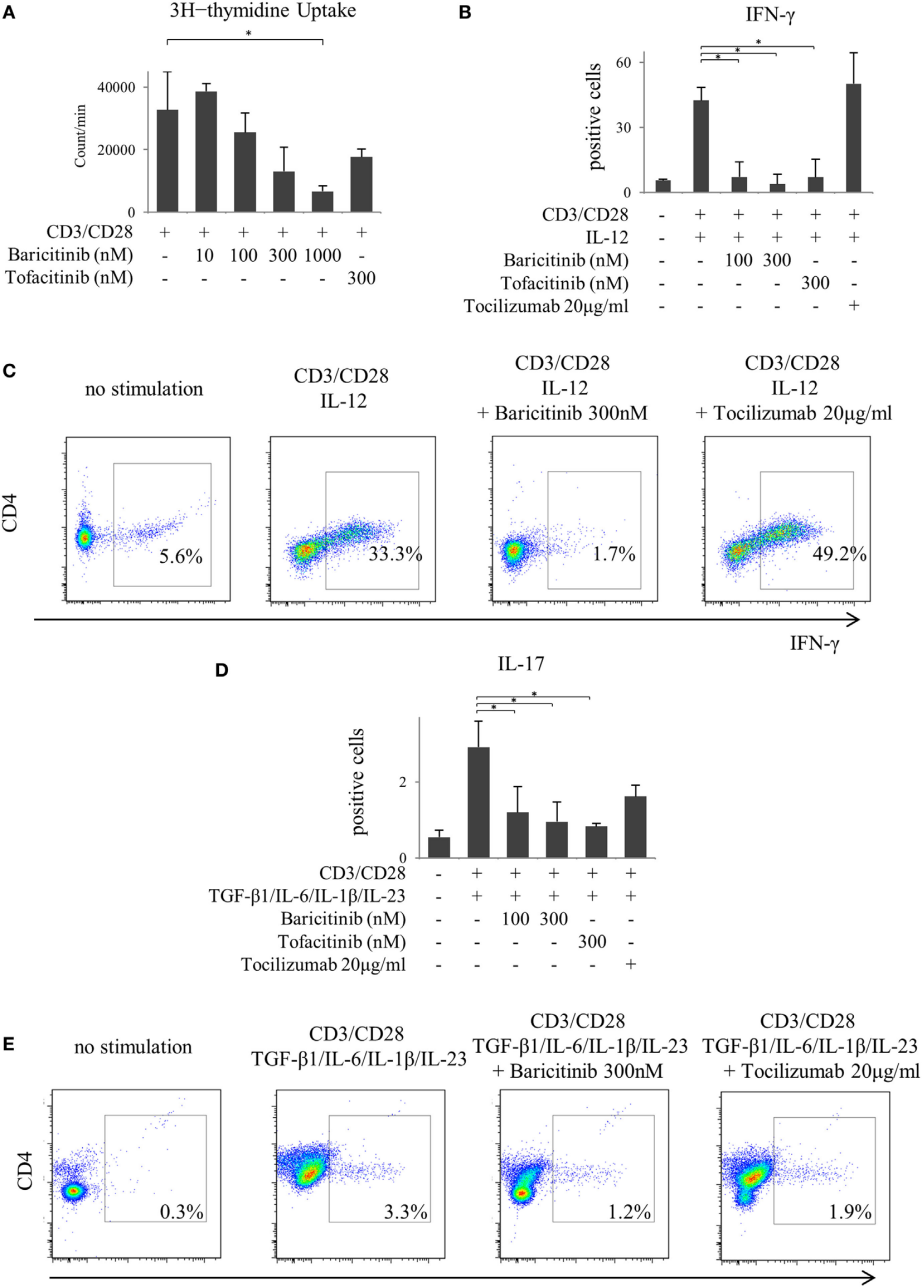
Baricitinib inhibits T cell proliferation and differentiation of Th1 and Th17 cells. Human naive CD4^+^ T cells were purified and cultured with anti-CD3 antibody, anti-CD28 antibody, and various cytokines [interleukin (IL)-12, TGF-β, IL-6, IL-1β, and IL-23]. **(A)** T cell proliferation assessed by [^3^H] thymidine incorporation. **(B)** Percentage of interferon (IFN)-γ-producing cells in T cells in the presence of baricitinib and other inhibitors. **(C)** Representative flow cytometry plots showing IFN-γ production in T cells. **(D)** Percentage of IL-17-producing cells in T cells in the presence of baricitinib and other inhibitors. **(E)** Representative flow cytometry plots showing IL-17 production in T cells. Data are mean ± SD of three different donors per group. *p < 0.05 (by Mann–Whitney *U* test).

Next, we investigated the actions of JAK inhibitors on T cells. Similar to other cells, baricitinib did not induce apoptosis of T cells (data not shown). Stimulation of CD3 and CD28 enhanced proliferation of CD4^+^ T cells. However, baricitinib inhibited the proliferation in a concentration-dependent manner (Figure 5A). A Similar effect was noted when tofacitinib was used. In the next step, we investigated CD4^+^ T cell differentiation. Since the results so far suggested the role of type-I IFN suppression, we explored the effect of type-I IFN on naïve CD4^+^ T cells. IFN-α did not enhance the IFN-γ and IL-17 productions from CD4^+^ T cells *in vitro* (data not shown). On the other hand, human naïve CD4^+^ T cells differentiated into Th1, which strongly produces IFN-γ at 5 days after IL-12 stimulation (Figures 5B,C). Baricitinib and tofacitinib, but not tocilizumab, inhibited this differentiation. Similarly, naïve CD4^+^ T cells differentiated into Th17, which produces IL-17, at 5 days after TGF-β1, IL-6, IL-1β, and IL-23 stimulation with anti-IFN-γ antibody (Figures 5D,E). Baricitinib inhibited this differentiation in a concentration manner. In addition to Th1 differentiation, this effect was reproduced by tofacitinib. Although tocilizumab also inhibited Th17 differentiation (*p* = 0.085), the effect was less than that produced by baricitinib (Figures 5D,E).

### Baricitinib Inhibits Phosphorylation of STAT Transcription Factors on T Cells

**Figure 6:**
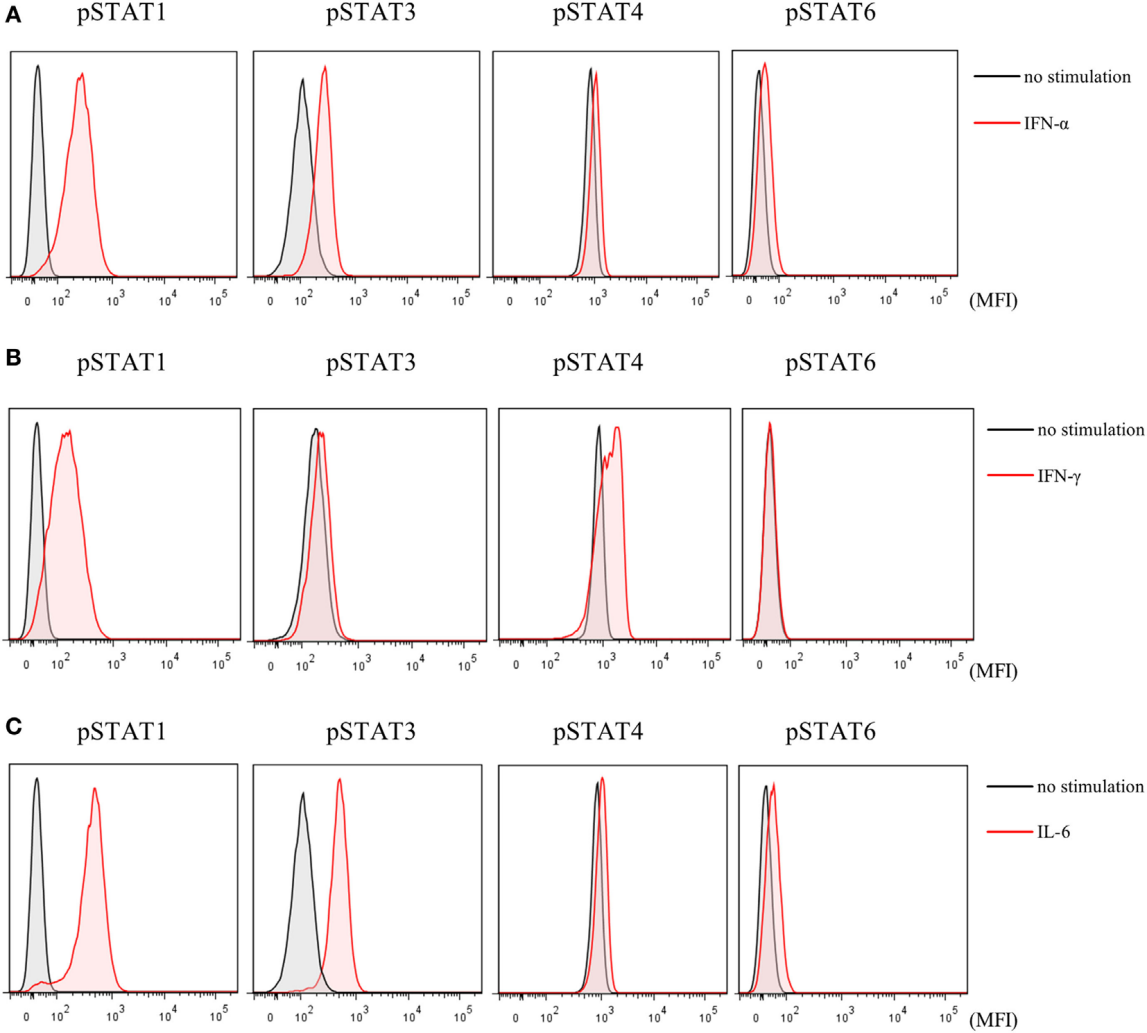
Phosphorylation of signal transducer and activator of transcription (STAT) mediated by type-I interferon (IFN), type 2 IFN, and interleukin (IL)-6. Representative flow cytometry plots. **(A)** Phosphorylation of STATs by IFN-α. **(B)** Phosphorylation of STATs by IFN-γ. **(C)** Phosphorylation of STATs by IL-6.

**Figure 7:**
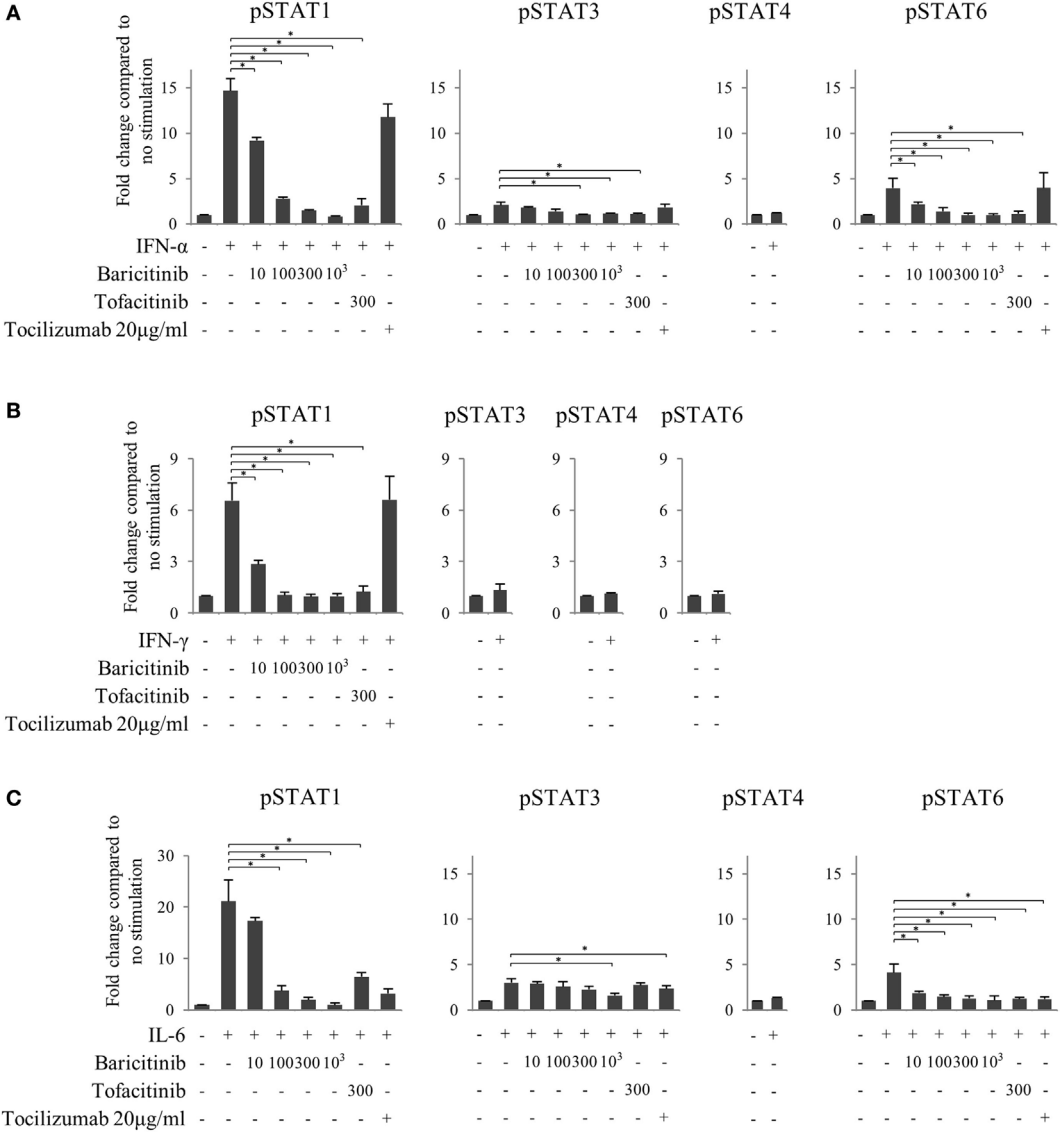
Baricitinib inhibits phosphorylation of signal transducer and activator of transcription (STAT) mediated by type-I interferon (IFN), type 2 IFN, and interleukin (IL)-6. Human naive CD4^+^ T cells were cultured in the presence of various cytokines (IFN-α, IFN-γ, and IL-6). **(A)** Expression of pSTATs mediated by IFN-α in the presence of baricitinib and other inhibitors. **(B)** Expression of pSTATs mediated by IFN-γ in the presence of baricitinib and other inhibitors. **(C)** Expression of pSTATs mediated by IL-6 in the presence of baricitinib and other inhibitors. Data are mean ± SD of three different donors per group. *p < 0.05 (by Mann–Whitney *U* test).

Finally, to investigate the effects of JAK inhibitors on the cytokine-mediated signaling pathway, we analyzed the effects of stimulation with IFN-α, IFN-γ, and IL-6 on STATs phosphorylation in human naive CD4^+^ T cells. IFN-α and IL-6 induced phosphorylation of STAT1 and STAT3, while IFN-γ only induced phosphorylation of STAT1 (Figures 6A–C). Although IFN-α and IL-6 showed numerically increased phosphorylation of STAT6 [based on mean fluorescence intensity (MFI)] and JAK inhibitors weakened this effect on MFI (Figures 7A–C), the numerical changes were considered to have meaningless biological importance since the differences in activity were small (Figures 6A–C).

Although both JAK inhibitors inhibited the phosphorylation of STATs induced by type-I IFN in a concentration-dependent manner, the magnitude of the suppression impact was similar in baricitinib and tofacitinib (Figure 7A). On the other hand, tocilizumab did not inhibit type-I IFN signal. Similarly, JAK inhibitors strongly inhibited type-II IFN-induced STAT1 phosphorylation, and the effects were similar in these two drugs (Figure 7B). These results could explain the similarity of IFN-mediated effects of baricitinib and tofacitinib on human immune cells. As expected, these effects were not seen with tocilizumab. Finally, all three inhibitors tested in this study (tocilizumab, baricitinib, and tofacitinib) suppressed IL-6 signal (Figure 7C).

## Discussion

This study was conducted to assess the effects of JAK inhibitors on human immune cells. The results demonstrated that JAK inhibitors can control various immune networks. In addition, to the best of our knowledge, this is the first study for elucidating the mode of action of baricitinib.

Clinical studies have reported the effectiveness of the selective JAK1/JAK2 inhibitor baricitinib in the treatment of RA (14–17). Tocilizumab is an antibody binding to the receptor for IL-6 signaling, which is transmitted *via* JAK1 and JAK2. Since tocilizumab is also clinically effective in patients with RA (23), inhibition of the IL-6/JAK1/JAK2 pathway is presumed to be one of the important mechanisms of actions of baricitinib in the treatment of RA. The results of this study also suggest that JAKs plays a significant role in human immune networks.

We reported previously that tofacitinib suppressed the T cell stimulatory capacity of DCs by suppressing costimulatory molecules (21). This phenomenon was explained by the inhibitory effect of tofacitinib on the autocrine feedback of type-I IFN, the receptor that binds JAK1, TYK2, and IRF7 (21). Our results confirmed that baricitinib can control innate immunity, similar to the effects of tofacitinib (21). In this study, assessment of the action of JAK inhibitors on pDCs, which are the main source of type-I IFN, showed suppressed production of type-I IFN, though the mechanism of this action was not elucidated. In general, when TLRs are stimulated by viral infection, pDCs are induced to produce minute amounts of IFNβ through IRF3 (24, 25). The produced IFN-β is activated in an autocrine manner and activates various transcription factors that in turn induce the expression of various subtypes of IFN-α (26–28). Baricitinib is assumed to inhibit type-I IFN production from pDCs by inhibiting the autocrine action of type-I IFN, as described in MoDCs (21). Although baricitinib completely inhibited the IFN-stimulated activation of STAT1, the effects on DCs were partial; baricitinib has almost no inhibitory effect on Tyk2, allowing persistent activity of the Tyk2 pathway for IRF7.

Meanwhile, from the point of view of the adaptive immunity, JAK inhibitors modulated the differentiation of B cells by suppressing signals from type-I IFN. Furthermore, the phenotype of T cells was associated with baricitinib-induced inhibition of IL-12 and IL-23. Since JAK2 and TYK2 are associated with the downstream signaling of both cytokines, inhibition of JAK2 seems to inhibit the differentiation of pathogenic helper T cells. This phenomenon was also observed with tofacitinib, confirming that tofacitinib inhibits JAKs (JAK1, JAK2, and JAK3), but not TYK2 (7). Thus, it seems there are no apparent differences between tofacitinib and baricitinib with regard to their effects on innate and adaptive immunity.

It might be unexpected that there were no differences between baricitinib and tofacitinib. As described above, this study demonstrated that the inhibition of JAK signaling leads to inhibition of type-I IFN signals and differentiation of pathogenic helper T cells. This suggests that JAK inhibitors can block various immune networks in autoimmune diseases, which is one of the most important findings of this study, and has the potential to be applied to the design of new therapeutic strategies for the treatment of various autoimmune diseases. These two drugs, which have clearly different a mode of action from biologics such as TNF inhibitors and anti-IL-6R antibody, play an important role in terms of IFN signature. Regarding the differences *in vivo*, the serum concentration of baricitinib seems to be higher than that of tofacitinib by single dose pharmacokinetics. However, we cannot compare the serum concentration between baricitinib and tofacitinib, since baricitinib is taken 4 mg once a day and tofacitinib is taken 5 mg twice a day. And, serum levels of approximately 100–300 nM seem to be achieved in the steady state for both drugs, although accurate concentration is not clearly informed. Meanwhile, there have been no reports comparing between tofacitinib and baricitinib, although both tofacitinib (6) and baricitinib (29) have ameliorated arthritis in murine models.

On the other hand, excessive blockade of the immune networks may results in unwanted side effects. It should be emphasized that the changes induced by JAK inhibitors in the present study were concentration dependent. This is because JAK inhibitors bind to the adenosine triphosphate-binding sites of JAK in a concentration-dependent manner to competitively inhibit it. In the context of treatment of RA, dose adjustment is currently difficult with bDMARDs, which are typically injected by the subcutaneous route at a pre-selected dose. In terms of the utility of oral dosing, although the simple administration route is often emphasized, it is also important to stress on the ease of dose adjustment.

In conclusion, this study demonstrated that JAK inhibitors affect innate and adaptive immunity in humans. They can fine-tune various immune networks through a variety of mechanisms and seem suitable potential therapeutic agents for the treatment of diverse autoimmune diseases.

## Ethics Statement

This study was reviewed and approved by the ethics committee of medical research, university of occupational and environmental health, Japan. PBMC were obtained from health volunteers. A signed informed consent was obtained from all subjects in accordance with the Declaration of Helsinki and its subsequent modifications.

## Author Contributions

SK contributed to the study design, overall review, and writing of the manuscript. The other authors were involved in the performance of the study and review of the manuscript. SN and YT participated in the study design, overall review, and coordination. All the authors read and approved the final manuscript.

## Conflict of Interest Statement

SK has received speaking fees from Bristol-Myers, Pfizer, and Takeda. SN has received speaking fees from Bristol-Myers, UCB, Astellas, Abbvie, Eisai, Pfizer, Takeda, and also research grants from Mitsubishi-Tanabe, Novartis, and MSD. KN has received speaking fees from UCB, Astellas, Mitsubishi-Tanabe, and research grants from Mitsubishi-Tanabe and Eisai. YT has received consulting fees, speaking fees, and/or honoraria from Abbvie, Daiichi-Sankyo, Chugai, Takeda, Mitsubishi-Tanabe, Bristol-Myers, Astellas, Eisai, Janssen, Pfizer, Asahi-kasei, Eli Lilly, GlaxoSmithKline, UCB, Teijin, MSD, and Santen, and also research grants from Mitsubishi-Tanabe, Takeda, Chugai, Astellas, Eisai, Taisho-Toyama, Kyowa-Kirin, Abbvie, and Bristol-Myers. KS and AI are employees of Mitsubishi-Tanabe Pharma. YK is an employee of Astellas. All the other authors declare no conflict of interest.

## References

[1] Smolen JS Aletaha D Bijlsma JW Breedveld FC Boumpas D Burmester G Treating rheumatoid arthritis to target: recommendations of an international task force. Ann Rheum Dis (2010) 69(4):631–7. 10.1136/ard.2009.123919 20215140 PMC3015099

[2] Smolen JS Breedveld FC Burmester GR Bykerk V Dougados M Emery P Treating rheumatoid arthritis to target: 2014 update of the recommendations of an international task force. Ann Rheum Dis (2016) 75(1):3–15. 10.1136/annrheumdis-2015-207524 25969430 PMC4717393

[3] Smolen JS Landewe R Bijlsma J Burmester G Chatzidionysiou K Dougados M EULAR recommendations for the management of rheumatoid arthritis with synthetic and biological disease-modifying antirheumatic drugs: 2016 update. Ann Rheum Dis (2017) 76(6):960–77. 10.1136/annrheumdis-2016-210715 28264816

[4] O’Shea JJ Plenge R. JAK and STAT signaling molecules in immunoregulation and immune-mediated disease. Immunity (2012) 36(4):542–50. 10.1016/j.immuni.2012.03.014 22520847 PMC3499974

[5] Karaman MW Herrgard S Treiber DK Gallant P Atteridge CE Campbell BT A quantitative analysis of kinase inhibitor selectivity. Nat Biotechnol (2008) 26(1):127–32. 10.1038/nbt1358 18183025

[6] Ghoreschi K Jesson MI Li X Lee JL Ghosh S Alsup JW Modulation of innate and adaptive immune responses by tofacitinib (CP-690,550). J Immunol (2011) 186(7):4234–43. 10.4049/jimmunol.1003668 21383241 PMC3108067

[7] Roskoski R Jr Janus kinase (JAK) inhibitors in the treatment of inflammatory and neoplastic diseases. Pharmacol Res (2016) 111:784–803. 10.1016/j.phrs.2016.07.038 27473820

[8] Fleischmann R Kremer J Cush J Schulze-Koops H Connell CA Bradley JD Placebo-controlled trial of tofacitinib monotherapy in rheumatoid arthritis. N Engl J Med (2012) 367(6):495–507. 10.1056/NEJMoa1109071 22873530

[9] Kremer J Li ZG Hall S Fleischmann R Genovese M Martin-Mola E Tofacitinib in combination with nonbiologic disease-modifying antirheumatic drugs in patients with active rheumatoid arthritis: a randomized trial. Ann Intern Med (2013) 159(4):253–61. 10.7326/0003-4819-159-4-201308200-00006 24026258

[10] van der Heijde D Tanaka Y Fleischmann R Keystone E Kremer J Zerbini C Tofacitinib (CP-690,550) in patients with rheumatoid arthritis receiving methotrexate: twelve-month data from a twenty-four-month phase III randomized radiographic study. Arthritis Rheum (2013) 65(3):559–70. 10.1002/art.37816 23348607

[11] van Vollenhoven RF Fleischmann R Cohen S Lee EB García Meijide JA Wagner S Tofacitinib or adalimumab versus placebo in rheumatoid arthritis. N Engl J Med (2012) 367(6):508–19. 10.1056/NEJMoa1112072 22873531

[12] Burmester GR Blanco R Charles-Schoeman C Wollenhaupt J Zerbini C Benda B Tofacitinib (CP-690,550) in combination with methotrexate in patients with active rheumatoid arthritis with an inadequate response to tumour necrosis factor inhibitors: a randomised phase 3 trial. Lancet (2013) 381(9865):451–60. 10.1016/S0140-6736(12)61424-X 23294500

[13] Kubo S Nakayamada S Tanaka Y. Baricitinib for the treatment of rheumatoid arthritis. Expert Rev Clin Immunol (2016) 12(9):911–9. 10.1080/1744666X.2016.1214576 27427830

[14] Genovese MC Kremer J Zamani O Ludivico C Krogulec M Xie L Baricitinib in patients with refractory rheumatoid arthritis. N Engl J Med (2016) 374(13):1243–52. 10.1056/NEJMoa1507247 27028914

[15] Dougados M van der Heijde D Chen YC Greenwald M Drescher E Liu J Baricitinib in patients with inadequate response or intolerance to conventional synthetic DMARDs: results from the RA-BUILD study. Ann Rheum Dis (2017) 76(1):88–95. 10.1136/annrheumdis-2016-210094 27689735 PMC5264214

[16] Fleischmann R Schiff M van der Heijde D Ramos-Remus C Spindler A Stanislav M Baricitinib, methotrexate, or combination in patients with rheumatoid arthritis and no or limited prior disease-modifying antirheumatic drug treatment. Arthritis Rheumatol (2017) 69(3):506–17. 10.1002/art.39953 27723271 PMC5347954

[17] Taylor PC Keystone EC van der Heijde D Weinblatt ME Del Carmen Morales L Reyes Gonzaga J Baricitinib versus placebo or adalimumab in rheumatoid arthritis. N Engl J Med (2017) 376(7):652–62. 10.1056/NEJMoa1608345 28199814

[18] Kubo S Nakayamada S Yoshikawa M Miyazaki Y Sakata K Nakano K Peripheral immunophenotyping identifies three subgroups based on T cell heterogeneity in lupus patients. Arthritis Rheumatol (2017) 69(10):2029–37. 10.1002/art.40180 28605137

[19] Banchereau R Hong S Cantarel B Baldwin N Baisch J Edens M Personalized immunomonitoring uncovers molecular networks that stratify lupus patients. Cell (2016) 165(3):551–65. 10.1016/j.cell.2016.03.008 27040498 PMC5426482

[20] Maeshima K Yamaoka K Kubo S Nakano K Iwata S Saito K The JAK inhibitor tofacitinib regulates synovitis through inhibition of interferon-γ and interleukin-17 production by human CD4+ T cells. Arthritis Rheum (2012) 64(6):1790–8. 10.1002/art.34329 22147632

[21] Kubo S Yamaoka K Kondo M Yamagata K Zhao J Iwata S The JAK inhibitor, tofacitinib, reduces the T cell stimulatory capacity of human monocyte-derived dendritic cells. Ann Rheum Dis (2014) 73(12):2192–8. 10.1136/annrheumdis-2013-203756 24013646

[22] Sato K Tsuchiya M Saldanha J Koishihara Y Ohsugi Y Kishimoto T Reshaping a human antibody to inhibit the interleukin 6-dependent tumor cell growth. Cancer Res (1993) 53(4):851–6. 8428365

[23] Smolen JS Beaulieu A Rubbert-Roth A Ramos-Remus C Rovensky J Alecock E Effect of interleukin-6 receptor inhibition with tocilizumab in patients with rheumatoid arthritis (OPTION study): a double-blind, placebo-controlled, randomised trial. Lancet (2008) 371(9617):987–97. 10.1016/S0140-6736(08)60453-5 18358926

[24] Yoneyama M Suhara W Fukuhara Y Fukuda M Nishida E Fujita T. Direct triggering of the type I interferon system by virus infection: activation of a transcription factor complex containing IRF-3 and CBP/p300. EMBO J (1998) 17(4):1087–95. 10.1093/emboj/17.4.1087 9463386 PMC1170457

[25] Sato M Tanaka N Hata N Oda E Taniguchi T. Involvement of the IRF family transcription factor IRF-3 in virus-induced activation of the IFN-beta gene. FEBS Lett (1998) 425(1):112–6. 10.1016/S0014-5793(98)00210-5 9541017

[26] Sato M Hata N Asagiri M Nakaya T Taniguchi T Tanaka N. Positive feedback regulation of type I IFN genes by the IFN-inducible transcription factor IRF-7. FEBS Lett (1998) 441(1):106–10. 10.1016/S0014-5793(98)01514-2 9877175

[27] Honda K Yanai H Negishi H Asagiri M Sato M Mizutani T IRF-7 is the master regulator of type-I interferon-dependent immune responses. Nature (2005) 434(7034):772–7. 10.1038/nature03464 15800576

[28] Honda K Taniguchi T. IRFs: master regulators of signalling by toll-like receptors and cytosolic pattern-recognition receptors. Nat Rev Immunol (2006) 6(9):644–58. 10.1038/nri1900 16932750

[29] Fridman JS Scherle PA Collins R Burn TC Li Y Li J Selective inhibition of JAK1 and JAK2 is efficacious in rodent models of arthritis: preclinical characterization of INCB028050. J Immunol (2010) 184(9):5298–307. 10.4049/jimmunol.0902819 20363976

